# Modern integrative prostate cancer diagnostics

**DOI:** 10.1097/MOU.0000000000001361

**Published:** 2025-12-10

**Authors:** Rainer Grobholz, Felice Burn, Lukas Prause

**Affiliations:** a Medical Faculty, University of Zurich, Zurich; b Institute of Pathology; c Institute of Radiology; d AI & Data Science CoE; e Department of Urology, Kantonsspital Aarau, Aarau, Switzerland

**Keywords:** artificial intelligence biomarker, artificial intelligence, digital pathology, magnetic resonance imaging, prostate cancer

## Abstract

**Purpose of review:**

To review contemporary applications, performance, and implementation challenges of artificial intelligence (AI) in the radiological and pathological diagnosis of prostate cancer, and to highlight emerging multimodal AI biomarkers for prognosis and treatment selection.

**Recent findings:**

In radiology, large multicenter studies demonstrate that MRI-based AI can detect clinically significant prostate cancer with accuracy comparable to, and in some contexts surpassing, expert radiologists, while reducing inter-reader variability and improving workflow efficiency. In surgical pathology, AI systems show high concordance with pathologists in cancer detection and Gleason grading, helping standardize challenging features such as Gleason pattern 4 and supporting triage or second-reader workflows. However, emerging transformative potential lies in multimodal AI systems that integrate digital histopathology with clinical and molecular data to deliver prognostic and predictive biomarkers. These tools are now being validated in randomized trials and real-world cohorts and are beginning to be recognized in clinical guidelines.

**Summary:**

AI is a powerful assistive technology that can enhance diagnostic accuracy, reproducibility, and efficiency across MRI and pathology. The integration of multimodal data is catalyzing validated biomarkers to guide risk stratification and treatment decisions – the next frontier in personalized prostate cancer care. But broad adoption still requires rigorous external validation, quality assurance, and ongoing postdeployment monitoring.

## INTRODUCTION

Although advancing rapidly in research, artificial intelligence (AI) in prostate cancer diagnostics remains investigational in most clinical settings. A central challenge is distinguishing indolent from clinical significant prostate cancer (csPCa) while minimizing unnecessary biopsies and overtreatment. Clinical practice has evolved from a linear prostate specific antigen (PSA) → biopsy pathway toward layered strategies that combine imaging, biomarkers, genomics, and especially AI to answer two practical questions: who truly needs a biopsy, and who benefits from treatment intensification. In this paradigm, multiparametric magnetic resonance imaging (mpMRI) and systematic plus targeted biopsies form the backbone of diagnosis, while digital pathology enables reproducible grading and quantification. AI has the potential to enhance these workflows as an assistive, reader-in-the-loop technology – standardizing image interpretation, reducing inter-reader variability, flagging suspicious lesions, and streamlining reporting workflows in both radiology and pathology. Early clinical evaluations and pilot deployments suggest that well validated, locally integrated and calibrated tools can improve efficiency without compromising diagnostic accuracy. Nevertheless, rigorous external validation, calibration, and postdeployment monitoring remain essential. This review summarizes current applications and evidence for MRI- and pathology-based AI and highlights the emerging role of multimodal AI biomarkers that integrate imaging with clinical variables to guide prognosis and treatment selection. 

**Box 1:**
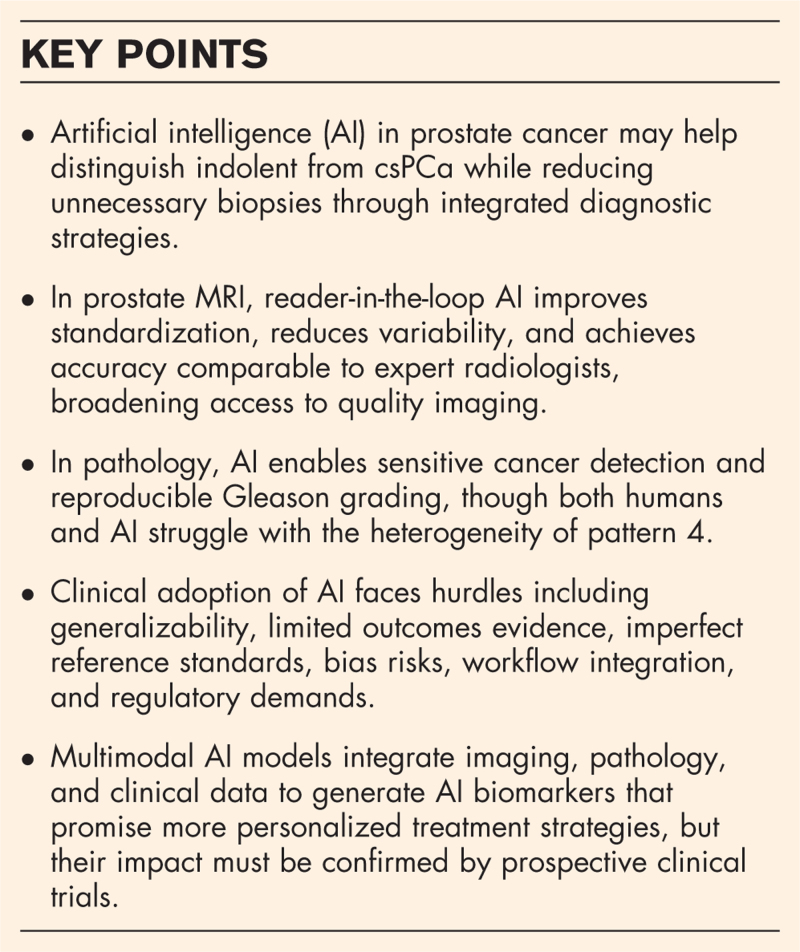
no caption available

## ARTIFICIAL INTELLIGENCE AND MRI

MRI before biopsy is recommended by major guidelines and increasingly implemented for biopsy-naïve men. This approach improves csPCa detection and reduces overdiagnosis by enabling targeted biopsies and decreases unnecessary procedures [1^▪^,2^▪^]. Examinations should be acquired and reported in accordance with the Prostate Imaging–Reporting and Data System (PI-RADS) v2.1 to ensure reproducible quality and facilitate clear multidisciplinary communication across centers. AI has emerged as a promising adjunct in prostate MRI diagnostics because it addresses several longstanding challenges. The interpretation of mpMRI and the PI-RADS scoring is subject to inter-reader variability and depends heavily on radiologist expertise. Reader-in-the-loop AI can help standardize assessments, reduce inter-reader variability, and improve reproducibility [1^▪^]. Moreover, prostate MRI interpretation is time-consuming. Reader-in-the-loop AI can preanalyze images, flag suspicious lesions, and provide lesion-level suspicion scores aligned with PI-RADS categories. This functionality reduces workload and accelerates reporting when AI is used as a triage tool or as a second reader [1^▪^,^3^]. Landmark evidence, most notably the Prostate Imaging: Cancer AI (PI-CAI) study, demonstrates that MRI AI achieves detection performance comparable to, and in some settings exceeding, experienced urogenital radiologists for csPCa [4^▪▪^]. Importantly, AI support can broaden access to high-quality MRI interpretation in community hospitals and resource-limited regions where experienced urogenital radiologists may be scarce. Collectively, these capabilities make AI an appealing technology to improve accuracy, reproducibility, efficiency, and equity of access in prostate cancer diagnostics.

Most MRI AI models are supervised deep learning systems (e.g., convolutional neuronal networks) trained on mpMRI or biparametric MRI (bpMRI) data with histopathology as the reference standard. These models typically rely on pipelines with extensive preprocessing and augmentation to ensure robustness. After training, they undergo external validation across multiple centers to assess generalizability, discrimination, calibration, and clinical utility [1^▪^,^3^]. In 2025, the PI-RADS Steering Committee outlined specific recommendations for AI development and reporting in biopsy-naïve men. Key requirements include dataset transparency, rigorous external validation, confirmation of true negatives through adjudication or follow-up, and adherence to the Checklist for Artificial Intelligence in Medical Imaging (CLAIM), providing a standardized protocol for implementers [1^▪^,^3^]. Clinical deployment must also comply with evolving regulations, including the EU AI Act (Regulation (EU) 2024/1689) and the U.S. Food and Drug Administration's (FDA) Predetermined Change Control Plan (PCCP) guidance for AI-enabled medical device software [5,6].

## LANDMARK PERFORMANCE EVIDENCE

The PI-CAI study represents an international benchmarking initiative on bpMRI using standardized hidden validation/testing cohorts. Its confirmatory study was trained on 9207 MRI examinations from three Dutch centers (11 sites) and tested on 1000 examinations from four centers (12 sites) in the Netherlands and Norway. Under controlled conditions, the best AI achieved area under the receiver operating characteristic curve (AUROC) was 0.91 compared with 0.86 for pooled radiologists (PI-RADS v2.1). At matched specificity (57.7%), AI detected 6.8% more csPCa; at matched sensitivity (89.4%), AI produced markedly fewer false positives and fewer Gleason grade group 1 diagnoses, supporting deployment as a triage or second reader rather than as an autonomous replacement [4^▪▪^]. The challenge deliberately focused on bpMRI (noncontrast) and, by releasing a large public dataset and competitive benchmark, catalyzed model development optimized for streamlined clinical workflows. Recent publications increasingly prioritize bpMRI and several report that AI-assisted bpMRI can, under prespecified protocols and in selected cohorts, achieve performance comparable to radiologist-interpreted mpMRI for csPCa, though findings remain context-dependent [7].

## READER STUDY OF BIPARAMETRIC MRI IN ROUTINE PRACTICE

In a diagnostic, multireader, multicase study 61 readers (34 experts, 27 nonexperts) from 53 centers in 17 countries assessed bpMRI examinations both without and with AI assistance [8]. The observer set comprised 360 examinations (from 780 men within PI-CAI), while the AI system was recalibrated on an independent set of 420 examinations to generate lesion detection maps (the recalibration set was not shown to readers) [8]. Disease presence was confirmed by histopathology and at least three years of follow-up. The primary endpoint was csPCa diagnosis, evaluated by AUROC with sensitivity and specificity at a PI-RADS threshold ≥3. With AI assistance, performance improved by approximately 3.3% AUROC (0.882 → 0.916), with consistent gains across both experts and nonexperts (with a more significant benefit for the nonexpert group), reinforcing day-to-day assistive value in noncontrast protocols [8].

## OUTSTANDING HURDLES TO CLINICAL ADOPTION

Despite rapid progress, several barriers must be addressed before prostate MRI AI can be fully integrated into routine practice:

(1) Outcomes evidence: Even large confirmatory studies focus on diagnostic accuracy rather than downstream clinical benefit [4^▪▪^].
(2) Generalization: Real-world performance remains fragile across centers, vendors, and protocols, depending critically on standardized acquisition and quality control (e.g., PI-QUAL v2) [9,10].
(3) Reference standards: Biopsy under-samples disease and MRI–histopathology registration introduces error, creating label noise that hampers robust training and benchmarking [11].
(4) Reader variability: Substantial inter-reader variability in PI-RADS complicates algorithm development, external validation, and fair head-to-head comparisons with radiologists [12].
(5) Fairness and shortcuts: Models can infer sensitive attributes (e.g., patient race) from images, raising concerns about hidden bias [13].
(6) Human factors: Automation bias and workflow integration issues may limit efficiency and safety gains [14].
(7) Regulation, privacy, economics: Evolving regulations (EU AI Act; FDA PCCP), stringent de-identification of DICOM data, and uncertain cost-benefit ratios necessitate prospective monitoring, calibration, and change control [5,6,15,16].

## CURRENT RESEARCH: TOWARDS A “DIGITAL BIOPSY”

Recently, Shao *et al.* introduced the MRI-based Predicted Transformer for Prostate Cancer (MRI-PTPCa) model, which combines self-supervised contrastive pretraining with a transformer architecture that fuses multisequence MRI and multitask heads for csPCa detection, Gleason grade group prediction, and lesion localization. The model emphasizes interpretability (attention heatmaps) and robustness to missing diffusion-weighted imaging/apparent diffusion coefficient [17^▪▪^]. Trained and tested across seven Chinese centers and prospectively evaluated as a standalone “red flag” system, MRI-PTPCa achieved an AUROC of 0.983 for any prostate cancer and of 0.978 for Gleason grade groups 2–5, with 89.1% grading accuracy. Crucially, it outperformed diagnostic biopsies in predicting the final Gleason grade group in radical prostatectomies and surpassed PI-RADS scoring (AUROC of 0.98 vs. 0.75 for Gleason grade groups 2–5) [17^▪▪^]. On the PI-CAI public cohort (*n* = 1500), MRI-PTPCa reported an AUROC of 0.972 for Gleason grade groups 2–5; for context, the PI-CAI confirmatory study's top 5 ensemble achieved an AUROC of 0.93 on the hidden 1000-case test set—an across-cohort/protocol comparison that still underscores state-of-the-art performance [4^▪▪^,^17^^▪▪^].

## ARTIFICIAL INTELLIGENCE IN SURGICAL PATHOLOGY

The increasing incidence of prostate cancer diagnoses in an aging global population, combined with heightened disease awareness, has led to a substantial rise in prostate biopsies [18]. Prostate core needle biopsies remain the standard diagnostic modality in histopathology. However, the associated workload has expanded considerably as case volumes increase. Updated international guidelines, such as those from the International Society of Urologic Pathology [19] and the Genitourinary Pathology Society [20], have further intensified this demand by requiring detailed reporting of tumor length, percentage involvement, perineural invasion, and the proportion of Gleason pattern (GP) 4 for every biopsy core.

AI-based histological assessment has demonstrated diagnostic accuracy comparable to expert pathologists and offers significant potential for standardized, efficient pathology reporting. Key advantages include rapid analysis, integration into routine diagnostic workflows, preprocessing of digital slides, and potential use for automated triage or second-opinion consultation. Clinical implementation of such systems could optimize workload distribution and improve diagnostic reliability, particularly in routine cases where efficiency is paramount. Several studies have demonstrated the robustness and reliability of AI algorithms for prostate cancer detection and grading [21–25,26^▪^]. Across these studies, AI reliably distinguished prostate cancer from benign tissue and provided Gleason grading highly comparable to that of experienced pathologists. A comprehensive meta-analysis further confirmed high diagnostic accuracy for prostate cancer detection, with sensitivities exceeding 90% in most studies (87–100%) and specificities ranging from 68% to 99% [26^▪^]. In addition, Raciti *et al.* reported that general pathologists, when supported by AI, improved their sensitivity by 8% and their specificity by 0.7% [27]. These findings highlight how AI's capacity to analyze digitized slides with high sensitivity and specificity is reshaping diagnostic workflows, enabling rapid screening of large tissue volumes, highlighting suspicious foci, and even producing preliminary Gleason grades for pathologist review (Fig. 1 a–c).

**FIGURE 1:**
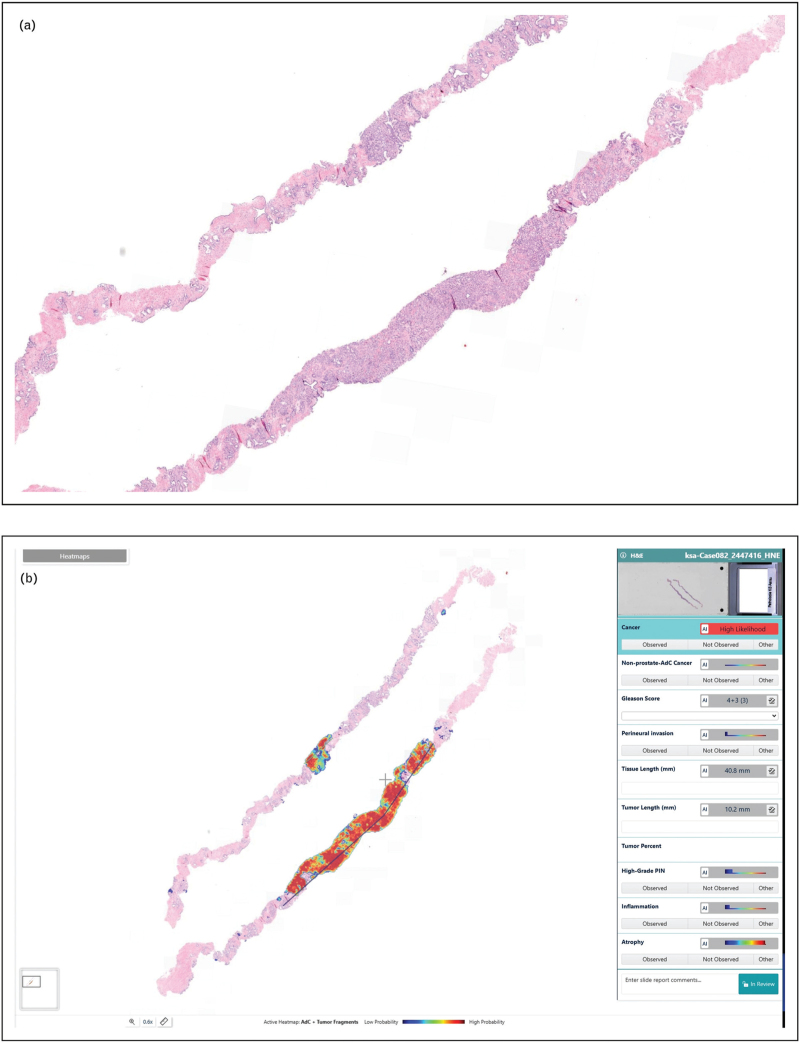
Detection of prostate cancer and Gleason score assignment. Example of an acinar adenocarcinoma in two core needle biopsies, analyzed by an AI algorithm (IBEX Galen Prostate AI). (a) Hematoxylin & Eosin stained tissue. (b) Heat map of detected tumor areas and measured tumor length. (c) Heat map of the detected Gleason patterns, suggesting a Gleason score 4+3 = 7, Gleason grade group 3.

**FIGURE 1 (Continued):**
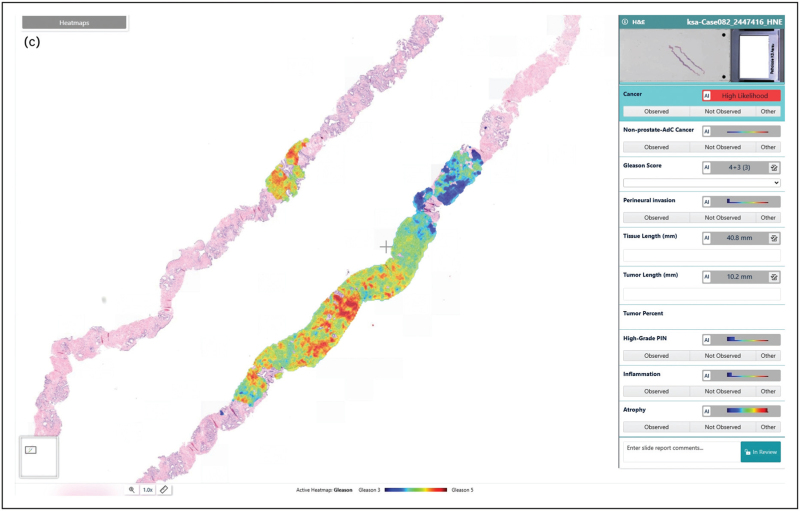
Detection of prostate cancer and Gleason score assignment. Example of an acinar adenocarcinoma in two core needle biopsies, analyzed by an AI algorithm (IBEX Galen Prostate AI). (a) Hematoxylin & Eosin stained tissue. (b) Heat map of detected tumor areas and measured tumor length. (c) Heat map of the detected Gleason patterns, suggesting a Gleason score 4+3 = 7, Gleason grade group 3.

However, true real-world validation is hampered because commercial algorithms rarely undergo transparent testing on public benchmark datasets, and data from clinical studies often remain unavailable to the academic community. To address this, a recent study curated a diverse and challenging dataset of 113 whole-slide prostate biopsy images from seven sources using global crowdsourcing of pathologists. The dataset emulated the variability of actual clinical settings, including different imaging scanners, staining protocols, and the presence of artifacts [28^▪▪^]. Ten expert pathologists, most subspecialized in uropathology, participated in a reader study, collectively grading all cases. Gleason grading predictions were also solicited from five public algorithms and two commercial products (Paige and AIRA Matrix). Both public and commercial AI algorithms showed high overall concordance with pathologists, measured by quadratic weighted kappa: Pathologists’ pairwise agreement ranged 0.777–0.916, algorithms ranged 0.617–0.900. Commercial algorithms matched or outperformed academic algorithms, pathologists excelled at excluding benign cases (higher specificity), while AI algorithms were particularly sensitive in tumor identification.

Despite these advances, the intrinsic complexity and subjectivity of Gleason grading remain a major challenge. In particular, the accurate identification of GP4 is difficult both for human pathologists and for AI systems. In a study of 6185 images from 297 patients, AI demonstrated strong concordance with experts for GP3 and GP5 but only moderate agreement for GP4. The detection of GP4 was shown to be magnification-dependent, with superior accuracy at higher resolutions (20× objective), while GP3 and GP5 were more consistently recognized across magnifications [29^▪▪^]. However, in some cases, AI can miss comedonecrosis (which clearly indicates a GP5 pattern) and falsely sign out as GP4 pattern (Fig. 2 , own observation). Even with targeted training – including exposure to the full morphological spectrum of GP4 – the AI algorithm struggled to capture the marked heterogeneity of this pattern. This limitation closely paralleled the substantial variability observed among human pathologists, reaffirming GP4 as the principal source of grading discordance. Moreover, an important observation from this study was that quantitative analysis revealed a consistent underestimation of GP4 by human observers, with pathologists typically sizing GP4 lesions at approximately half their true extent [29^▪▪^]. The latter underlines the influence of cognitive and perceptual constraints in Gleason grading, particularly for GP4, and emphasizes AI's potential for providing objective, scalable, and reproducible quantification.

**FIGURE 2:**
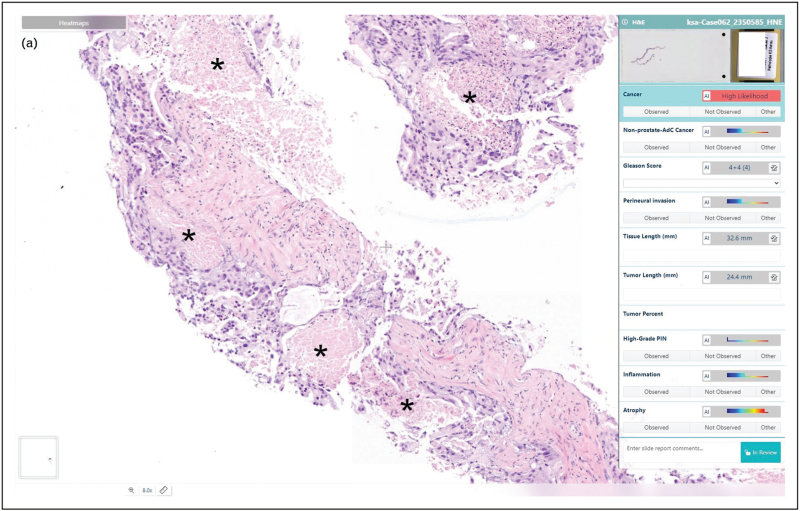
Difficulties in assigning Gleason patterns by AI. (a) Example of a poorly differentiated acinar adenocarcinoma with comedonecrosis (asterisks) indicating a Gleason pattern 5, evaluated by two different AI algorithms. (b) Areas of comedonecrosis are not detected, and therefore assigned as Gleason pattern 4 (IBEX Galen Prostate AI). (c) Areas of comedonecrosis are not detected, and therefore assigned as Gleason pattern 4 (Cancercenter.ai, Gleason Score 4.0).

**FIGURE 2 (Continued):**
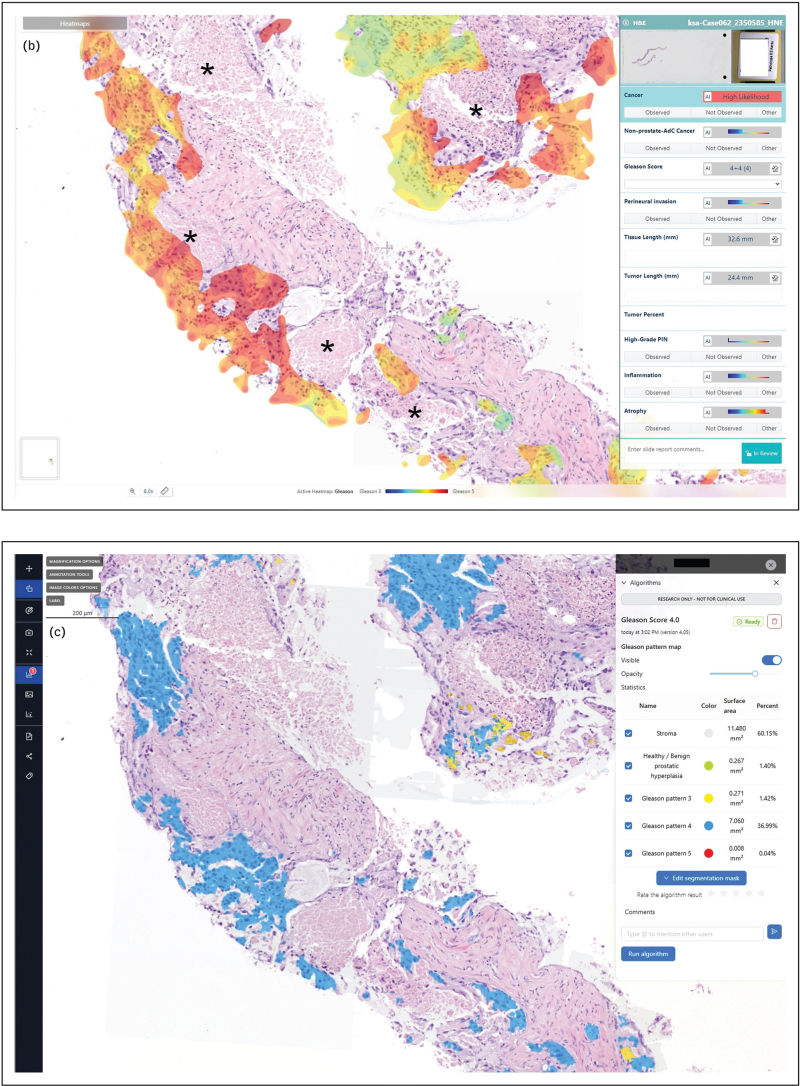
Difficulties in assigning Gleason patterns by AI. (a) Example of a poorly differentiated acinar adenocarcinoma with comedonecrosis (asterisks) indicating a Gleason pattern 5, evaluated by two different AI algorithms. (b) Areas of comedonecrosis are not detected, and therefore assigned as Gleason pattern 4 (IBEX Galen Prostate AI). (c) Areas of comedonecrosis are not detected, and therefore assigned as Gleason pattern 4 (Cancercenter.ai, Gleason Score 4.0).

The digitization of pathology has enabled both improved tissue pattern recognition and advanced biomarker discovery. By leveraging molecular, cellular, and clinical datasets, machine learning (ML) and deep learning (DL) models can identify complex, nonintuitive biological patterns of significance. These so-called AI biomarkers—features within biological or medical data whose clinical relevance emerges through artificial intelligence analysis—represent a rapidly evolving domain [30^▪^].

Multimodal artificial intelligence (MMAI) approaches advance this field by integrating diverse data types, including digital histopathology images and structured clinical variables, to generate powerful predictive biomarkers for prognosis and therapy response in prostate cancer [31]. MMAI models are trained by merging DL-derived image features with key clinical variables, such as the National Comprehensive Cancer Network (NCCN) risk factors, age, PSA, and Gleason pattern annotations, culminating in individualized risk stratification tools capable of predicting outcomes including distant metastasis, biochemical recurrence, and survival.

A prominent example is a recently developed MMAI model, which combines digital pathology with clinical metrics to guide risk stratification in localized prostate cancer. This system has undergone clinical validation in Phase 3 randomized trials and real-world datasets, offering prognostic scores for long-term outcomes and facilitating patient selection for hormone therapies and advanced treatment regimens [31–33,34,35^▪▪^]. This MMAI model has further demonstrated predictive validity for overall survival in metastatic hormone-sensitive disease cohorts, retaining robust performance even when controlling for treatment modalities and disease burden. Retrospective studies corroborate significant associations between MMAI scores and critical endpoints, such as biochemical recurrence and adverse pathology postradical prostatectomy [35]. Reflecting this clinical impact, this MMAI model by ArteraAI has recently been incorporated as an advanced risk stratification tool in the NCCN guidelines (V2.2026) and has received de novo marketing authorization from the FDA in August 2025. However, FDA has given permission to use or market the product under specific conditions, but the product has not met the full approval standard yet. Prospective studies confirming its clinical value are still pending.

## CONCLUSION

Artificial intelligence is increasingly transforming both radiology and pathology in prostate cancer diagnostics, evolving from proof-of-concept applications to early clinical implementations in daily routine. In prostate MRI, AI tools are most mature when deployed in assistive, reader-in-the-loop workflows. Applications such as triage and second-reader systems have demonstrated the capacity to reduce inter-reader variability, highlight overlooked lesions, standardize reporting, and shorten interpretation times. These developments not only improve diagnostic reproducibility but may also expand access to high-quality imaging in community and resource-limited settings, provided that local calibration, PI-QUAL–based quality assurance, and ongoing performance monitoring are ensured. In pathology, AI has shown high sensitivity for cancer detection, consistency in Gleason grading, and potential for workload optimization, with particular promise in resolving areas of high diagnostic variability such as Gleason pattern 4 assessment. While prospective, large-scale validation studies remain essential, early evidence indicates that integration of AI into clinical workflows can enhance both efficiency and diagnostic accuracy. Multimodal approaches that integrate imaging, pathology, clinical, and molecular data represent the emerging frontier, enabling AI-derived capable of guiding individualized prognosis and therapy. With the recent FDA authorization and guideline inclusion of the Artera MMAI model, the field is moving toward a transformative paradigm in which AI augments precision, efficiency, and personalization in prostate cancer management.

## Acknowledgements

*None.*

### Financial support and sponsorship

*None.*

### Conflicts of interest

*There are no conflicts of interest.*

## REFERENCES AND RECOMMENDED READING

Papers of particular interest, published within the annual period of review, have been highlighted as:

▪ of special interest
▪▪ of outstanding interest

## References

[1▪] Padhani AR Papanikolaou N. AI and human interactions in prostate cancer diagnosis using MRI. Eur Radiol 2025; 35:5695–5700. 40055229 10.1007/s00330-025-11498-0

[2▪] Ng AB Giganti F Kasivisvanathan V. Artificial intelligence in prostate cancer diagnosis on magnetic resonance imaging: time for a new PARADIGM. Eur Urol 2025; 88:4–7. 40312251 10.1016/j.eururo.2025.04.018

[3] Alis D Onay A Colak E. A narrative review of artificial intelligence in mri-guided prostate cancer diagnosis: addressing key challenges. Diagnostics (Basel) 2025; 15:1342. 40506914 10.3390/diagnostics15111342 PMC12154491

[4▪▪] Saha A Bosma JS Twilt JJ. Artificial intelligence and radiologists in prostate cancer detection on MRI (PI-CAI): an international, paired, noninferiority, confirmatory study. Lancet Oncol 2024; 25:879–887. 38876123 10.1016/S1470-2045(24)00220-1 PMC11587881

[5] European Union. Regulation (EU) 2024/1689 of the European Parliament and of the Council of 13 June 2024 laying down harmonised rules on Artificial Intelligence (AI Act). 2024; https://eur-lex.europa.eu/eli/reg/2024/1689/oj/eng [Accessed 2025/10/03].

[6] U.S. Food and Drug Administration. Marketing submission recommendations for a predetermined change control plan for artificial intelligence enabled device software functions: guidance for industry and FDA staff. 2025; https://www.fda.gov/regulatory-information/search-fda-guidance-documents/marketing-submission-recommendations-predetermined-change-control-plan-artificial-intelligence [Accessed 2025/10/03].

[7] Nissler D Reimers-Kipping S Ingwersen M. Artificial intelligence-assisted biparametric MRI for detecting prostate cancer – a comparative multireader multicase accuracy study. J Clin Med 2025; 14:6111. 40943871 10.3390/jcm14176111 PMC12429428

[8] Twilt JJ Saha A Bosma JS. AI-assisted vs unassisted identification of prostate cancer in magnetic resonance images. JAMA Netw Open 2025; 8:e2515672. 40512493 10.1001/jamanetworkopen.2025.15672 PMC12166490

[9] Giganti F Moreira da SN Yeung M. AI-powered prostate cancer detection: a multicentre, multiscanner validation study. Eur Radiol 2025; 35:4915–4924. 40016318 10.1007/s00330-024-11323-0 PMC12226644

[10] de Rooij M Allen C Twilt JJ. PI-QUAL version 2: an update of a standardised scoring system for the assessment of image quality of prostate MRI. Eur Radiol 2024; 34:7068–7079. 38787428 10.1007/s00330-024-10795-4 PMC11519155

[11] Singh S Mathew M Mertzanidou T. Histo-MRI map study protocol: a prospective cohort study mapping MRI to histology for biomarker validation and prediction of prostate cancer. BMJ Open 2022; 12:e059847. 10.1136/bmjopen-2021-059847 PMC8995953 35396316

[12] Wen J Ji Y Han J. Inter-reader agreement of the prostate imaging reporting and data system version v2.1 for detection of prostate cancer: a systematic review and meta-analysis. Front Oncol 2022; 12:1013941. 36248983 10.3389/fonc.2022.1013941 PMC9554626

[13] Gichoya JW Banerjee I Bhimireddy AR. AI recognition of patient race in medical imaging: a modelling study. Lancet Digit Health 2022; 4:e406–e414. 35568690 10.1016/S2589-7500(22)00063-2 PMC9650160

[14] Dratsch T Chen X Rezazade MM. Automation bias in mammography: the impact of artificial intelligence BI-RADS suggestions on reader performance. Radiology 2023; 307:e222176. 37129490 10.1148/radiol.222176

[15] Kondylakis H Catalan R Alabart SM. Documenting the de-identification process of clinical and imaging data for AI for health imaging projects. Insights Imaging 2024; 15:130. 38816658 10.1186/s13244-024-01711-x PMC11139818

[16] Brat HG. Cutting through the hype: the true economic impact and ROI of AI in radiology. Eur Radiol 2024; 34:7904–7906. 38922449 10.1007/s00330-024-10873-7

[17▪▪] Shao L, Liang C, Yan Y, *et al.* An MRI-pathology foundation model for noninvasive diagnosis and grading of prostate cancer. Nat. Cancer 2025; 6:1621–1637. 10.1038/s43018-025-01041-x 40897909

[18] Vaccarella S Li M Bray F. Prostate cancer incidence and mortality in Europe and implications for screening activities: population based study. BMJ 2024; 386:e077738. 39231588 10.1136/bmj-2023-077738 PMC11372856

[19] van Leenders GJLH van der Kwast TH Grignon DJ. The 2019 International Society of Urological Pathology (ISUP) consensus conference on grading of prostatic carcinoma. Am J Surg Pathol 2020; 44:e87–e99. 32459716 10.1097/PAS.0000000000001497 PMC7382533

[20] Epstein JI Amin MB Fine SW. The 2019 Genitourinary Pathology Society (GUPS) white paper on contemporary grading of prostate cancer. Arch Pathol Lab Med 2021; 145:461–493. 32589068 10.5858/arpa.2020-0015-RA

[21] Pantanowitz L Quiroga-Garza GM Bien L. An artificial intelligence algorithm for prostate cancer diagnosis in whole slide images of core needle biopsies: a blinded clinical validation and deployment study. Lancet Digit Health 2020; 2:e407–e416. 33328045 10.1016/S2589-7500(20)30159-X

[22] Eloy C Marques A Pinto J. Artificial intelligence-assisted cancer diagnosis improves the efficiency of pathologists in prostatic biopsies. Virchows Arch 2023; 482:595–604. 36809483 10.1007/s00428-023-03518-5 PMC10033575

[23] Steiner DF Nagpal K Sayres R. Evaluation of the use of combined artificial intelligence and pathologist assessment to review and grade prostate biopsies. JAMA Netw Open 2020; 3:e2023267. 33180129 10.1001/jamanetworkopen.2020.23267 PMC7662146

[24] Bulten W Balkenhol M Belinga JA. Artificial intelligence assistance significantly improves Gleason grading of prostate biopsies by pathologists. Mod Pathol 2021; 34:660–671. 32759979 10.1038/s41379-020-0640-y PMC7897578

[25] Marginean F Arvidsson I Simoulis A. An artificial intelligence-based support tool for automation and standardisation of gleason grading in prostate biopsies. Eur Urol Focus 2021; 7:995–1001. 33303404 10.1016/j.euf.2020.11.001

[26▪] Morozov A Taratkin M Bazarkin A. A systematic review and meta-analysis of artificial intelligence diagnostic accuracy in prostate cancer histology identification and grading. Prostate Cancer Prostatic Dis 2023; 26:681–692. 37185992 10.1038/s41391-023-00673-3

[27] Raciti P Sue J Retamero JA. Clinical validation of artificial intelligence-augmented pathology diagnosis demonstrates significant gains in diagnostic accuracy in prostate cancer detection. Arch Pathol Lab Med 2023; 147:1178–1185. 36538386 10.5858/arpa.2022-0066-OA

[28▪▪] Faryna K Tessier L Retamero J. Evaluation of artificial intelligence-based gleason grading algorithms “in the Wild”. Mod Pathol 2024; 37:100563. 39025402 10.1016/j.modpat.2024.100563

[29▪▪] Eminaga O Abbas M Kunder C. Critical evaluation of artificial intelligence as a digital twin of pathologists for prostate cancer pathology. Sci Rep 2024; 14:5284. 38438436 10.1038/s41598-024-55228-w PMC10912767

[30▪] Alum EU. AI-driven biomarker discovery: enhancing precision in cancer diagnosis and prognosis. Discov Oncol 2025; 16:313. 40082367 10.1007/s12672-025-02064-7 PMC11906928

[31] Markowski MC Ren Y Tierney M. Digital pathology-based artificial intelligence biomarker validation in metastatic prostate cancer. Eur Urol Oncol 2025; 8:755–762. 39665917 10.1016/j.euo.2024.11.009 PMC12369405

[32] Esteva A Feng J van der Wal D. Prostate cancer therapy personalization via multimodal deep learning on randomized phase III clinical trials. NPJ Digit Med 2022; 5:71. 35676445 10.1038/s41746-022-00613-w PMC9177850

[33] Spratt DE Tang S Sun Y. Artificial intelligence predictive model for hormone therapy use in prostate cancer. NEJM Evid 2023; 2:EVIDoa2300023. 38320143 10.1056/EVIDoa2300023 PMC11195914

[34▪▪] Parker CTA Mendes L Liu VYT. External validation of a digital pathology-based multimodal artificial intelligence-derived prognostic model in patients with advanced prostate cancer starting long-term androgen deprivation therapy: a posthoc ancillary biomarker study of four phase 3 randomised controlled trials of the STAMPEDE platform protocol. Lancet Digit Health 2025; 7:100885. 40467357 10.1016/j.landig.2025.100885

[35] Wang JH Deek MP Mendes AA. Validation of an artificial intelligence-based prognostic biomarker in patients with oligometastatic castration-sensitive prostate cancer. Radiother Oncol 2025; 202:110618. 39510141 10.1016/j.radonc.2024.110618 PMC11663099

